# Within-Subject Associations between Mood Dimensions and Non-exercise Activity: An Ambulatory Assessment Approach Using Repeated Real-Time and Objective Data

**DOI:** 10.3389/fpsyg.2016.00918

**Published:** 2016-06-24

**Authors:** Markus Reichert, Heike Tost, Iris Reinhard, Alexander Zipf, Hans-Joachim Salize, Andreas Meyer-Lindenberg, Ulrich W. Ebner-Priemer

**Affiliations:** ^1^Department of Psychiatry and Psychotherapy, Central Institute of Mental Health, Medical Faculty Mannheim, Heidelberg UniversityMannheim, Germany; ^2^Department of Sports and Sports Science, Karlsruhe Institute of TechnologyKarlsruhe, Germany; ^3^Division of Biostatistics, Central Institute of Mental Health, Medical Faculty Mannheim, Heidelberg UniversityMannheim, Germany; ^4^GIScience Research Group, Institute of Geography, Heidelberg UniversityHeidelberg, Germany; ^5^Department of Psychosomatic Medicine and Psychotherapy, Central Institute of Mental HealthMannheim, Germany

**Keywords:** ambulatory assessment, ecological momentary assessment, mood, affective states, physical activity, non-exercise activity, activities of daily life, accelerometry

## Abstract

A physically active lifestyle has been related to positive health outcomes and high life expectancy, but the underlying psychological mechanisms maintaining physical activity are rarely investigated. Tremendous technological progress yielding sophisticated methodological approaches, i.e., ambulatory assessment, have recently enabled the study of these mechanisms in everyday life. In practice, accelerometers allow to continuously and objectively monitor physical activity. The combination with e-diaries makes it feasible to repeatedly assess mood states in real-time and real life and to relate them to physical activity. This state-of-the-art methodology comes with several advantages, like bypassing systematic distortions of retrospective methods, avoiding distortions seen in laboratory settings, and revealing an objective physical activity assessment. Most importantly, ambulatory assessment studies enable to analyze how physical activity and mood wax and wane within persons over time in contrast to existing studies on physical activity and mood which mostly investigated between-person associations. However, there are very few studies on how mood dimensions (i.e., feeling well, energetic and calm) drive non-exercise activity (NEA; such as climbing stairs) within persons. Recent reviews argued that some of these studies have methodological limitations, e.g., scarcely representative samples, short study periods, physical activity assessment via self-reports, and low sampling frequencies. To overcome these limitations, we conducted an ambulatory assessment study in a community-based sample of 106 adults over 1 week. Participants were asked to report mood ratings on e-diaries and to wear an accelerometer in daily life. We conducted multilevel analyses to investigate whether mood predicted NEA, which was defined as the mean acceleration within the 10-min interval directly following an e-diary assessment. Additionally, we analyzed the effects of NEA on different time frames following the e-diary prompts in an exploratory manner. Our results revealed that valence significantly and positively predicted NEA within persons (*p* = 0.001). Feeling more energetic was associated with significantly increased NEA (*p* < 0.001), whereas feeling calmer was associated with significantly decreased NEA (*p* < 0.001) on the within-person level. The analyses on different time frames of NEA largely confirmed our findings. In conclusion, we showed that mood predicted NEA within adults but with distinct magnitudes and directions of effects for each mood dimension.

## Introduction

Physical activity is an important determinant of human health, and being physically active has been shown to contribute to the prevention of and recovery from severe somatic and psychiatric diseases (for an overview refer to Pedersen and Saltin, 2015). Specifically, both engaging in exercise (such as swimming, playing football, working out at the gym) and living a physically active life comprising high levels of non-exercise activity (NEA; e.g., daily routines of walking to the office, biking for transport, climbing stairs) have been related to high health expectancy, for example reductions in the risk of cardiovascular disease (Healy et al., 2008; Wen et al., 2011). A better understanding of the psychological determinants of physical activity is of central significance to enhancing health promoting lifestyles with less sedentary behavior. According to behavioral theories, affective experiences play an important role in human behavior. In other words, activities related to positive affective experiences are supposed to be more likely to be repeated than those associated with a negative mood.

Although a large body of intervention studies have investigated between-subject effects of exercise on mood (for an overview refer to Reed and Ones, 2006; Reed and Buck, 2009), unfortunately, there has been a lack of research on the within-subject associations between physical activity and mood. Between-subject designs do not reveal how physical activity and mood wax and wane within people over time. Therefore, the between-subject findings that people who are on average more active than others feel better on average than others provide no evidence that there is a within-subject timely relationship between both of those processes, i.e., that within subjects, episodes of activity are related to good moods and that episodes of low activity are related to bad moods. From a more theoretical perspective, between-subject associations cannot be translated into within-subject relationships (Aarts et al., 2014), which implies that if the topic of interest is how mood maintains physical activity, a within-subject assessment approach is mandatory.

To investigate within-subject associations of physical activity and mood in participants' everyday lives, ambulatory assessment is currently the most promising and recommended state-of-the-art technique (Kanning et al., 2015). The use of e-diaries on smartphones to repeatedly assess mood states in real-time and of accelerometers to continuously and objectively monitor activity provides several advantages. It enables researchers to (a) assess the dynamic and time-dependent interplay between mood and physical activity (Ebner-Priemer and Trull, 2009); (b) assess mood in real time and thus bypass the systematic distortions observed in retrospective methods (Stone et al., 2002; Fahrenberg et al., 2007); (c) avoid the distortions produced by laboratory settings (Bussmann et al., 2009); and (d) increase the validity of physical activity assessments compared with participant self-ratings (Prince et al., 2008; Adamo et al., 2009).

Over the past decade, several research groups have used ambulatory assessment approaches to study within-subject effects of physical activity on mood in everyday life. Schwerdtfeger et al. (2008) found higher intensity and/or duration of everyday life activity to be associated with higher positive activated affect in 124 healthy participants over 12 assessment-hours. Significant positive effects of physical activity on energetic arousal and a significant negative effect on calmness were shown by Kanning et al. (2012) in a sample of 44 undergraduates over 24 h. In 2013, Bossmann et al. showed significant positive effects of physical activity on energetic arousal and valence in 77 university students over the course of 24 h. Kanning (2013) showed physical activity to be positively related with subsequent ratings of valence and energetic arousal as well as negative effects of physical activity on calmness in a sample of 87 undergraduates. In 2015, Kanning et al. revealed significantly heightened energetic arousal and more agitation (calmness) following physical activity over 3 days in adults. Dunton et al. (2015) found social interaction and physical context (i.e., being outdoors) to moderate the effects of physical activity on mood.

Although the abovementioned studies show increasing evidence for physical activity affecting mood within subjects and a large body of treatment studies have found effects of exercise on mood (for a discussion we refer to Schlicht et al., 2013), the within-subject impact of mood on physical activity remains largely unknown. This is surprising because psychomotor retardation has been conceptualized as a consequence of major depressive disorder ever since and many studies showed limited physical activity in depressed patients (Burton et al., 2013; Reichert et al., 2015). Thus, research in this direction seems very promising.

Unfortunately, only very few within-subject studies have investigated the predictive value of mood on non-exercise activity (Liao et al., 2015). In 2007, Carels et al investigated the associations of mood and exercise in a sample of 36 obese adults who participated in a behavioral weight loss program. Mood was assessed every morning and every evening as well as prior to and after each bout of exercise over at least 4 weeks within the weight loss program. Both mood and exercise data (i.e., duration, intensity and type) were collected via participants' self-reports in diaries. Carels et al. focused on the investigation of mood-exercise associations and therefore did not take non-exercise activity into account. Regarding the effects of mood on exercise, Carels et al. (2007) found mood in the morning to significantly increase the initiation of exercise on the same day both on the within- and between-person level. However, mood assessed in the morning was not related to either the duration or the intensity of exercise.

Dunton et al. (2009) reported associations between affective factors and non-exercise activity in 23 physically inactive adults aged 50 years and above. The adults were asked to complete electronic diaries in the morning, at midday, in the afternoon and in the evening to report on positive/negative affect, energetic arousal and types of non-exercise activity, among others. Dunton et al. (2009) found an increase in positive affect and a decrease in negative affect at *t*–1 to be associated with significantly increased moderate-to-vigorous physical activity at the following assessment (*t*). However, the trend in the affect dimension of energetic arousal, i.e., feeling more energetic and less tired being related to enhanced levels of non-exercise activity, lacked significance. Dunton et al. (2014) conducted an ambulatory assessment study to investigate whether mood does affect physical activity in 119 children aged 11 to 13 years. They analyzed how mood ratings on e-diaries influenced accelerometer-measured moderate-to-vigorous physical activity within the 30 min following the prompts. Although increased levels of energy and decreased ratings of fatigue were associated with increases in moderate-to-vigorous physical activity, the positive and negative affect ratings were not related to subsequent physical activity.

Schwerdtfeger et al. (2010) analyzed the effects of mood on physical activity across a 12-h period in a sample of 126 adults mainly comprising students. In their study, Personal Digital Assistants (PDAs) randomly queried for mood approximately 12 times, and physical activity was assessed using accelerometers attached to participant's ankles. Schwerdtfeger et al. (2010) parameterized physical activity by applying the categories sedentary, light, moderate, and vigorous activity based on thresholds using unit counts. Moreover, they averaged the different time frames of physical activity following mood assessments, namely 1 min, 1 to 5 min, 1 to 15 min, and 1 to 30 min. Positive activated affect was significantly associated with physical activity across all aggregated time frames, i.e., the more the participants felt good and energized, the more they were physically active. In addition, low negative affect significantly predicted higher physical activity within the 15 and 30 min following a mood assessment. In addition, Schwerdtfeger et al. (2010) found mood to influence different intensity levels of physical activity, showing an inverse association between enhanced mood and sedentary periods. In other words, higher positive activated affect and lower negative affect led to less sedentary behavior. Heightened positive activated affect and diminished negative affect predicted moderate to vigorous physical activity as well.

Wichers et al. (2012) analyzed the associations between self-rated physical activity and mood in 504 female twins who filled out booklets when triggered by a digital wrist watch approximately 10 times per day throughout a study period of 5 days. Their hypotheses were mainly focused on the effects of increases in physical activity on mood. Interestingly, their data suggested that increases in physical activity followed a decrease in positive affect. However, this finding showed significance only for the positive affect that was rated three prompts prior to the increase in physical activity. Moreover, the finding could not be replicated in both groups of twins. Mata et al. (2012) compared the associations of physical activity and mood between 53 depressed patients and healthy controls. Over 7 days, the participants rated mood and physical activity on palmtops approximately 8 times per day. Mata et al. (2012) found positive affect to increase before bouts of physical activity and to decrease afterwards but no effects of negative affect on physical activity in both depressed and healthy participants. This finding implies a peak of positive affect at the time participants were physically active. However, as Mata et al.'s study (2012) was based on self-reported physical activity, the exact time points of activity are not known, and therefore suggestions of the exact temporal course of physical activity and mood remain unclear.

To sum up, the results of these studies suggest that an increase in positive affect within subjects is followed by enhanced physical activity in everyday life, whereas negative affect does not necessarily predict physical activity; feeling more energetic and less tired may increase physical activity. However, these findings are limited and inconclusive (for a discussion refer to Liao et al., 2015). Moreover, the abovementioned studies had methodological limitations.

In detail, Liao et al. (2015) criticized (a) the convenient recruiting strategies such as studying volunteers from a university, (b) the short study periods of a few hours or days, and (c) the rare use of accelerometers to objectively assess physical activity, i.e., only in two studies accelerometers were used (Schwerdtfeger et al., 2010; Dunton et al., 2014) whereas the other four studies were based on participant self-reports. This previous point is particularly important as self-reported physical activity has been shown to be only modestly related to objectively assessed physical activity (Prince et al., 2008; Adamo et al., 2009). Thus, Liao et al. (2015) identified the need for (a) investigations of representative community-based samples, (b) long assessment periods exceeding at least 1 day, and (c) assessments using accelerometers and e-diaries and reports on missing data. Moreover, Schwerdtfeger et al. (2010) raised (d) the issue of temporal resolution, i.e., investigating how mood and physical activity are interconnected over time. Unfortunately, in previous studies, mood was mostly assessed via self-report, and therefore it was impossible to investigate the associations with a high temporal resolution. Specifically, when assessing mood and physical activity only at 8 time points during the day, for example, time-lagged analyses remain very limited due to the large temporal offset between mood ratings and physical activity reports. However, these investigations are important to garner a deeper understanding of how mood affects physical activity over time, e.g., in which time frames mood influences physical activity.

To investigate how mood is associated with subsequent non-exercise activity and to overcome the methodological limitations of the very few existing studies on this issue, we conducted an ambulatory assessment study (a) in a large community-based sample of adults (b) over the course of 1 week. We (c) objectively assessed non-exercise activity (via accelerometer) and queried mood repeatedly (i.e., valence, energetic arousal and calmness) in real time and real life (using electronic diaries on smartphones). To investigate (d) which time frames of physical activity are associated with the mood-dimensions valence, energetic arousal and calmness, we conducted explorative analyses.

We hypothesized a positive relationship between both mood dimensions, valence and energetic arousal, and subsequent non-exercise activity. Given the temporal order, this does imply that valence (hypothesis I) and energetic arousal (hypothesis II) would predict non-exercise activity. Moreover, we hypothesized that calmness was associated with subsequent non-exercise activity (hypothesis III). As the effects of the mood dimension of calmness on physical activity have not yet been investigated, we stated a nondirectional hypothesis.

## Materials and methods

### Participants

The sample for the current analyses was derived from the ongoing URGENCY study (Impact of Urbanicity on Genetics, Cerebral Functioning and Structure and Condition in Young People) implemented at the Central Institute of Mental Health in Mannheim (CIMH), Germany. The psychiatric-epidemiological center (PEZ) at the CIMH was responsible for recruiting participants aged between 18 and 28 years. The current sample was drawn from the municipalities of Mannheim, Ludwigshafen, Heidelberg, Weinheim, and the Rhine-Neckar district containing parts of the Forest of Odes in the period from December 2014 to September 2015. Monetary compensation was provided for participation in the URGENCY study. Participants with moderate to severe impairment of intelligence, participants unable to provide consent or with legal incapacity and participants with acute diseases were excluded. Additional exclusion criteria were a lifetime history of cardiovascular, chronic endocrine, immunological, or clinically manifested mental disorders. To enable the exclusion of exercise periods and thereby focus our analyses on non-exercise activity, only data from participants who reported on their exercise activities within the study period were included. Of the initial 106 participants' datasets, 9 were excluded for missing accelerometer data (lost devices, incomplete recordings, etc.). Furthermore, 3 datasets were excluded because of the substantial amount of time that the accelerometer was not worn or because the e-diary compliance was below 30%. One dataset was additionally excluded because of the shifted diurnal rhythm of the participant resulting from shift work. Finally, the analyzed sample comprised 93 participants (62.4% female) with a mean age of 23.4 years (*SD* = 2.7); their mean BMI was 22.8 kg/m^2^ (*SD* = 3.6).

### Ambulatory assessment procedure

First, participants were instructed on how to use the smartphone (Motorola Moto G^1^) and accelerometer (Move-II^2^) during an extensive briefing consisting of individual device tests at the PEZ. Thereafter, they carried both devices in their everyday life for seven consecutive days. Mood was assessed every day from 7:30 to 22:30, applying a mixed sampling strategy implemented on the Android smartphones using the experience sampling software movisensXS (version 0.6.3658)^2^.

In practice, participants were prompted every 40 to 100 min from 7:30 to 22:30 by an acoustic, vibration and visual signal on the smartphone; that is, they were invited to complete e-diary ratings at least 9 and up to 22 times per day. E-diary prompts could be postponed for 5, 10 or 15 min. In detail, e-diary assessment triggers were GPS-based and time-based. Since previous investigations revealed that standard approaches (e.g., time-based sampling) are likely to miss episodes of high non-exercise activity which occur rarely in everyday life (Ebner-Priemer et al., 2013), we used GPS-triggered e-diaries to optimize the sampling strategy (Dorn et al., 2015). Specifically, assessments were triggered every time the participants covered distances above 0.5 km. Moreover, the time-based sampling initiated prompts at fixed times (8:00 and 22:20) and additionally at least every 100 min from the preceding prompt (time-out trigger); it inhibited triggers within 40 min of the preceding prompt.

We aimed to focus the analyses on non-exercise activity. Hence, when the participants returned the devices at the PEZ after their study week, they were asked to report on their performed bouts of exercise within the assessment period (i.e., exercise duration, point in time) to enable the exclusion of these time frames from the analyses. To optimize participants' recall, we applied an approach following the idea of the Day Reconstruction Method (Kahneman et al., 2004). In practice, participants were shown the locations where they had stayed within the study period (tracked via smartphone) on a map. The participants were asked to label the locations (such as at home, at work, in the park, in the gym) to facilitate the recollection of exercise activities.

### Measures

Non-exercise activity was assessed using the triaxial acceleration sensors of the move-II^2^ (measurement range: +/–8 g; sampling frequency: 64 Hz; resolution: 12 bits; storage: internal memory card). Participants attached the devices at their right hip during the day for the entire study period except when sleeping. To assess mood, a short scale comprising two bipolar items for the mood dimensions valence, energetic arousal, and calmness, respectively, was administered in the e-diaries. This instrument was based on the Multidimensional Mood Questionnaire (Steyer et al., 1997) and was developed and evaluated by Wilhelm and Schoebi (2007) for the purposes of ambulatory assessment studies. They showed good psychometric properties on the between-person level with reliability coefficients of 0.92 for valence (items: unwell to well, discontent to content) and 0.90 for both energetic arousal (items: energy to full energy, tired to awake) and calmness (items: tense to relaxed, agitated to calm) and coefficients of 0.70 (valence and calmness) and 0.77 (energetic arousal) for the within-person level. Hence, this short scale was appropriate to assess within-person dynamics of mood over time in everyday life on e-diaries. In our study, visual analogue scales (0–100) were used. Additionally, the items were presented in reversed polarity and in a mixed order, as recommended by Wilhelm and Schoebi (2007).

### Analyses

First, we processed the accelerometer data computing Movement Acceleration Intensity [milli-g/min], i.e., the mean value of acceleration captured at the three sensor axis applying vector addition, using the software DataAnalyzer^2^. With this step, gravitational components were excluded using a high-pass filter (0.25 Hz), and artifacts (such as shocks to the device from driving by car) were excluded using a low-pass filter (11 Hz).

Second, we combined the e-diary data and the 1-min intervals of Movement Acceleration Intensity [milli-g] using the software DataMerger (version 1.6.3868)^2^. Based on former studies (e.g., Bossmann et al., 2013; Kanning, 2013; Kanning et al., 2015), we parameterized non-exercise activity (such as walking to the supermarket, being physically active at work, etc.) as the aggregated mean Movement Acceleration Intensity [milli-g] across 10-min time frames.

Third, we used SPSS (IBM; version 23.0.0.0) to aggregate mean Movement Acceleration Intensity [milli-g] for the 10-min intervals directly following each e-diary entry (later referred to as [0–10]). Because we were also interested in the time course of effects of mood dimensions on non-exercise activity, we aggregated mean Movement Acceleration Intensity [milli-g] within consecutive 10-min intervals following each e-diary entry. Specifically, we aggregated mean Movement Acceleration Intensity [milli-g] for the time frames from 11–20, 21–30, …, up to 281–290, and 291–300 min (later referred to as [11–20], [21–30], etc.). Moreover, to focus our analyses on non-exercise activity and to thus not take exercise (such as jogging, playing tennis, etc.) into account, we excluded e-diary-assessments that were collected within 300 min prior to participants' exercises for all analyses. Additionally, because the distribution of non-exercise activity showed only few very high values and was right-skewed (due to a high amount of the participants' sedentary time), we log-transformed all outcome variables for the purposes of statistical analyses. Specifically, all computed values for the intervals of non-exercise activity ([0–10], [11–20], [21–30],…, up to [291–300]) were log-transformed applying the natural logarithm.

**Figure 1:**
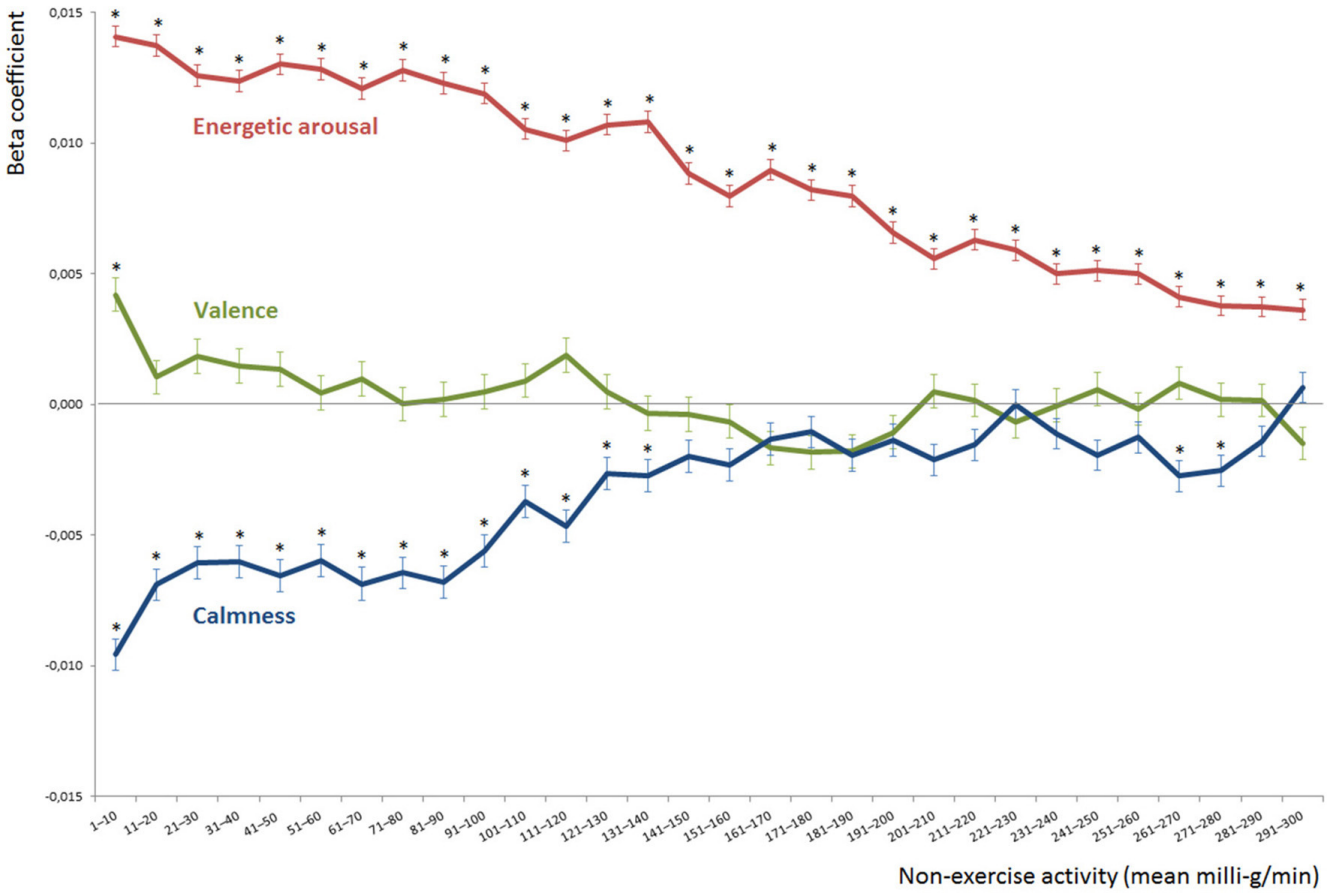
**Within-subject effects of mood dimensions on consecutive 10-min intervals of non-exercise activity**. The y-axis shows the beta coefficients for valence, energetic arousal and calmness predicting the non-exercise activity occurring in the consecutive 10-min intervals after the e-diary prompts. The 10-min intervals of non-exercise activity are displayed on the x-axis, e.g., the axis label [41–50] refers to the mean non-exercise activity from minute 41 up to minute 50 after the e-diary prompts. * show significant effects of valence, energetic arousal and calmness predicting 10-min intervals of non-exercise activity (p ≤ 0.05).

Thereafter, we conducted multilevel analyses to investigate within-person influences of mood dimensions (i.e., valence, energetic arousal and calmness) on non-exercise activity using SPSS (version 23.0.0.0., IBM). We nested repeated measurements (level 1) within participants (level 2) and calculated unconditional models to estimate intra-class correlations. Next, we calculated our main model using the outcome variable non-exercise activity, which was defined as the mean Movement Acceleration Intensity (logarithmized values) over the 10-min interval directly following the mood assessments. We included the level 1 predictors time, time-squared, valence within-subject, energetic arousal within-subject, and calmness within-subject. The predictors valence within-subject, energetic arousal within-subject, and calmness within-subject were centered around the persons‘ mean mood scores across the study week. The predictors time and time-squared were transformed to the daily study start time, i.e., we subtracted the value 7.5 because the study started at 7:30. To control for between-person effects, we added the level 2 predictors age, gender, BMI [kg/m^2^], and exercise/week [min]. Additionally, we added the mean mood scores for each participant as a level 2 predictor, namely the between-subject values for valence, energetic-arousal, and calmness. Specifically, these predictors were calculated by aggregating the mean mood scores across all e-diary assessments for the whole study week for each participant. We successively added random effects for each level 1 predictor. However, we retained only significant random effects in the final model (refer to Table 1). Finally, to investigate the short- and long-term impacts of mood on non-exercise activity, we calculated 30 multilevel models changing the outcome variable (refer to Figure 1). In detail, we used the aggregated mean Movement Acceleration Intensity (logarithmized values) across time frames [0–10], [11–20], [21–30], …, up to [291–300]. However, due to the results of our main model, we added only the level 2 predictors age, valence between-subject, energetic arousal between-subject, and calmness between-subject; we did not consider any random effects in these 30 models. We set the α-level to 0.05 for all the analyses.

**Table 1:** **Multilevel model analysis of influences of mood dimensions on non-exercise activity: Fixed and random effects**.

**Outcome**	**Fixed**				**Random**			
**Predictor**	**Beta coefficient**	**Standard Error**	***t*-Value (df)**	***p*-Value**	**Variance estimate**	***SD***	**Wald-Z**	***p*-value**
Intercept	2.60744	0.48976	5.32 (87.3)	<0.001	0.10377	0.01901	5.46	<0.001
Time [hours]	0.23052	0.01347	17.12 (5726.1)	<0.001				
Time-squared [hours^2^]	−0.01334	0.00087	−15.34 (5785.4)	<0.001				
Age [years]	−0.02416	0.01426	−1.69 (85.7)	0.094				
Gender	0.03948	0.08555	0.46 (85.9)	0.646				
BMI [kg/m^2^]	0.00480	0.01061	0.45 (83.4)	0.652				
Exercise/week [min]	−0.00022	0.00028	−0.79 (89.6)	0.434				
Valence within-subject	0.00444	0.00131	3.38 (5412.1)	0.001				
Energetic arousal within-subject	0.01411	0.00099	14.21 (96.3)	<0.001	0.00003	0.00001	2.25	0.025
Calmness within-subject	−0.01023	0.00166	−6.16 (123.0)	<0.001	0.00010	0.00003	3.28	0.001
Valence between-subject	0.02026	0.00762	2.66 (88.2)	0.009				
Energetic arousal between-subject	−0.00063	0.00480	−0.13 (88.0)	0.896				
Calmness between-subject	−0.01380	0.00604	−2.29 (85.7)	0.025				

#### Main model

Level 1 equation:

$$\begin{array}{l} Y_{ij}=\beta_{0j}+\beta_{1j}\times {\text{Time}}_{ij}+\beta_{2j}\times {\text{Time}}_{ij}^{2}+\beta_{3j}\times {\text{Valence}}_{ij}+\beta_{4j}\\ \times \;{\text{Energetic Arousal}}_{ij}+\beta_{5j}\times {\text{Calmness}}_{ij}+r_{ij} \end{array}$$

Level 2 equation:

$$\begin{array}{l} \beta_{0j}=\gamma_{00}+\gamma_{01}\times {\text{Age}}_{j}+\gamma_{02}\;\times {\text{Gender}}_{j}+\gamma_{03}\times {\text{BMI}}_{j}\\ +\gamma_{04}\times {\text{Exercise}}_{j}+\gamma_{05}\times {\text{Valence}}_{j}+\gamma_{06}\\ \times \;{\text{Energetic Arousal}}_{j}+\gamma_{07}\times {\text{Calmness}}_{j}+u_{0j}\\ \beta_{1j}=\gamma_{10}\\ \beta_{2j}=\gamma_{20}\\ \beta_{3j}=\gamma_{30}\\ \beta_{4j}=\gamma_{40}+u_{4j}\\ \beta_{5j}=\gamma_{50}+u_{5j} \end{array}$$

The effects within subjects were estimated at level 1. The subscript *j* refers to participant *j* and subscript *i* to the time of measurement. The level of non-exercise activity at time *i* in participant *j* is represented by *Y*_*ij*_. The intercept and the effects of time, time-squared, valence within-subject, energetic arousal within-subject, and calmness within-subject are represented by the beta coefficients (β) at level 1. The residuals are represented by *r*_*ij*_. The between-subject effects were estimated at level 2. As stated above, we retained only significant random effects in our final model. Since we found significant random effects only for the predictors energetic arousal within-subject and calmness within-subject, *u*_4*j*_ and *u*_5*j*_ represent the variation of participants' individual slopes for the predictors energetic arousal within-subject and calmness within-subject around the respective overall mean slope (refer to Table 1).

#### Model for analyses of time course

For the analyses on the short- and long-term impacts of mood on non-exercise activity (refer to Figure 1), we used the model presented below. We calculated 30 multilevel models, changing only the outcome variables. Specifically, we inserted the non-exercise activity within the time frames [0–10], [11–20], [21–30], …, up to [291–300] min after each e-diary entry as the outcome variables.

Level 1 equation:

$$\begin{array}{l} Y_{ij}=\beta_{0j}+\beta_{1j}\;\times {\text{Time}}_{ij}+\beta_{2j}\;\times {\text{Time}}_{ij}^{2}\;+\;\beta_{3j}\times {\text{Valence}}_{ij}+\beta_{4j}\\ \times \;{\text{Energetic Arousal}}_{ij}+\beta_{5j}\times {\text{Calmness}}_{ij}+r_{ij} \end{array}$$

Level 2 equation:

$$\begin{array}{l} \beta_{0j}=\gamma_{00}+\gamma_{01}\times {\text{Age}}_{j}+\gamma_{02}\times {\text{Valence}}_{j}+\gamma_{03}\\ \times \;{\text{Energetic Arousal}}_{j}+\gamma_{04}\times {\text{Calmness}}_{j}+u_{0j}\\ \beta_{1j}=\gamma_{10}\\ \beta_{2j}=\gamma_{20}\\ \beta_{3j}=\gamma_{30}\\ \beta_{4j}=\gamma_{40}\\ \beta_{5j}=\gamma_{50} \end{array}$$

To compute the percentage changes in non-exercise activity, we used the equation below.

$$\begin{array}{l} \delta =(e^{\beta (valence,\;energetic\;arousal,\;or\;calmness)\times 10})-\text{1})\times \text{100} \end{array}$$

### Ethical considerations

This study was approved by the ethics committee of the Medical Faculty Mannheim at the Ruprecht-Karls-University in Heidelberg. This study fulfilled the ethical guidelines for medical research according to the Declaration of Helsinki. Written and oral information regarding the study procedures were presented to all eligible participants before written informed consent was obtained. There was no surrogate consent procedure. All participants were free to withdraw from the study at any time.

## Results

### Descriptive statistics

The 93 participants completed 81.2% (*SD* = 14.3) of all e-diary prompts, i.e., on average 10 mood assessments/participant/day. To focus our analyses on the influences of mood on non-exercise activity, we excluded 9.2% of the completed e-diary entries that were followed by exercise within 300 min; the final data set consisted of a total of 5980 mood assessments. Participants' average non-exercise activity was 36.3 milli-g/participant/minute (range = [14.3–58.6]; *SD* = 9.8) across the 7 assessment days. For the sake of comparison, sedentary behavior (e.g., sitting) is associated with approximately 7 mg, walking (3.1 mph gait speed) with approximately 367 mg, and jogging (6.5 mph gait speed) with approximately 1103 mg (Anastasopoulou et al., 2014). The mean mood scores of the whole sample were 71.2 (*SD* = 10.5) for valence, 57.9 (*SD* = 10.6) for energetic arousal, and 67.7 (*SD* = 11.5) for calmness. The within-subject correlations between the mood components calculated applying Fisher's Z-transformation indicated that valence and energetic arousal were moderately associated *(r* = 0.36) across the study week. Moreover, valence and calmness were highly synchronized (*r* = 0.66) whereas energetic arousal and calmness were scarcely correlated *(r* = 0.08) within persons. Non-exercise activity was defined as the mean physical activity occurring in the 10-min intervals directly following the e-diary assessments. The intra-class correlation coefficient (ρ_*I*_ = 0.068) revealed that 93.2% of the variance was explained by within-subject variation in non-exercise activity. A total of 49 participants (52.7%) engaged in exercise, with an average exercise duration of 186.2 min/participant/week (*SD* = 137.8).

### Predicting non-exercise activity

Table 1 shows the influences of various within-person (level 1) and between-person (level 2) predictors on non-exercise activity. Non-exercise activity was parameterized into 10-min intervals of physical activity following each e-diary prompt. Descriptively, seven of twelve predictors showed significance, namely time (*p* < 0.001), time-squared (*p* < 0.001), valence within-subject (*p* = 0.001), energetic arousal within-subject (*p* < 0.001), calmness within-subject (*p* < 0.001), valence between subject (*p* = 0.009), and calmness between-subject (*p* = 0.025). Age, gender, BMI, energetic arousal between-subject, and exercise/week lacked significance, i.e., were not associated with non-exercise activity.

As expected, we found significant influences of the three mood dimensions on non-exercise activity within persons. Specifically, valence was positively related to the 10-min episodes of non-exercise activity following each e-diary assessment within-persons (refer to Table 1), thus confirming hypothesis I. However, the small beta coefficient (0.004) indicated a minor influence of valence on non-exercise activity. In practice, a 10-point increase in valence (on a scale from 0 to 100) led to an average increase in non-exercise activity of 4.5% within the 10 min after an e-diary assessment. Regarding the mood dimension of energetic arousal, we again found a significant positive influence on non-exercise activity (refer to Table 1), thus confirming hypothesis II. The beta coefficient of energetic arousal (0.014) was much higher than that of valence. In practice, when participants felt 10 points more energized (on a scale from 0 to 100), their non-exercise activity increased on average by 15.2% over the 10-min interval post-e-diary prompt.

As hypothesized (hypothesis III), calmness was also significantly related to non-exercise activity (refer to Table 1). The negative beta coefficient (−0.010) indicated that the calmer participants felt, the lower their subsequent non-exercise activity was. In practice, when participants felt 10 points (scale of 0 to 100) more calm, their non-exercise activity decreased on average by 9.7% in the 10 min after each e-diary prompt. We found significant random effects only for the within-subject predictors energetic arousal and calmness (refer to Table 1). This finding indicated between-subject variability in the within-subject relationship between mood dimensions and non-exercise activity. However, the variance estimates were minor (refer to Table 1), indicating negligible differences.

Moreover, our model showed that the participants' non-exercise activity was significantly influenced by the time of day, with positive effects of time (beta coefficient = 0.231) and negative effects of time-squared (beta coefficient = −0.013; refer to Table 1). Specifically, the effects of time on non-exercise activity were reversely u-shaped, i.e., non-exercise activity increased from the daily study start time (at approximately 7:30) to the afternoon (approximately 16:00) and then decreased until the study end time (at approximately 22:30). Additionally, we found significant between-person effects of valence and calmness on non-exercise activity (refer to Table 1). Participants who felt better on average across the study week showed on average higher non-exercise activity within the 10-min intervals following the e-diary assessments (beta coefficient = 0.020). Participants with heightened feelings of calmness throughout the study week revealed on average lower levels of non-exercise activity (beta coefficient = −0.014; refer to Table 1). The finding that participants' mean levels of valence and calmness across the study week were related to non-exercise activity was consistent with intervention studies showing positive between-subject effects of exercise on positive affect (for an overview refer to Reed and Ones, 2006; Reed and Buck, 2009). However, due to their between-subject designs, these studies did not investigate how physical activity and mood increased and decreased within people over time, i.e., they provided no evidence regarding whether there was a timely relationship between episodes of physical activity and mood within persons.

### Within-subject associations between mood dimensions and non-exercise activity over time

To obtain a deeper understanding of the short- and long-term impacts of mood on physical activity, we analyzed consecutive time frames of non-exercise activity to determine the effects of mood dimensions. Determination of these effects was previously requested by Schwerdtfeger et al. (2010), but the effects remained largely unknown due to the methodological limitations of former studies (Liao et al., 2015). Therefore, we conducted several multilevel-analyses using models that were similar to our full model presented above (refer to Table 1) and inserted consecutive 10-min time frames of non-exercise activity following the e-diary assessments as the outcome variable (11–20, 21–30, …, up to 281–290 and 291–300 min after e-diary assessment, later referred to as [11–20], [21–30], etc.). However, we excluded non-significant predictors (i.e., gender, BMI, exercise/week) and negligible random variables (i.e., within-subject energetic arousal and within-subject calmness) from the initial model. Figure 1 shows the influence of valence, energetic arousal and calmness on the consecutive 10-min intervals of average non-exercise activity after each e-diary prompt. Specifically, the y-axis depicts the beta coefficients of the mood dimensions influencing the respective 10-min interval of non-exercise activity, which is displayed on the x-axis. For example, the value of the red line on the y-axis for the time frame from [31–40] min reveals the direction (positive) and magnitude (beta coefficient = 0.012) of energetic arousal influencing non-exercise activity.

Figure 1 shows a significant influence of valence on non-exercise activity only within the [1–10] min following e-diary assessments (beta coefficient = 0.004; *p* = 0.001). However, across all averaged time frames thereafter, we found non-significant beta coefficients, with values close to zero (green line). Moreover, our models revealed significant effects of energetic arousal on non-exercise activity across all time frames up to 300 min, illustrated with the red line (refer to Figure 1). The beta coefficients depicting the influence of energetic arousal on non-exercise activity generally decreased from the time frame [1–10] (beta coefficient = 0.014; *p* < 0.001) to the time frame [291–300] (beta coefficient = 0.004; *p* < 0.001). Thus, the effects of energetic arousal were highest within the first 10-min interval directly following the e-diary prompts and then continuously decreased up to 300 min after the mood assessments. The fact that the beta coefficients approached zero was not surprising and demonstrates how the effects subsided over time. The significant beta coefficients with values noticeably larger than zero for all time frames up to 300 min fits nicely with our finding of a large influence of energetic arousal on non-exercise activity as determined in the main model (refer to Section Predicting Non-exercise Activity). Additionally, the mood dimension calmness again showed the greatest influence on the first 10-min interval directly following the mood assessment (beta coefficient = −0.010; *p* < 0.001). The following beta coefficients decreased up to 300 min after the e-diary prompts. The significant and noticeably non-zero beta coefficients up to the time frame of [131–140] min (beta coefficient = −0.003; *p* = 0.026) again fit nicely with our finding that calmness negatively influenced non-exercise activity (refer to Section Predicting Non-exercise Activity).

## Discussion

As expected, our analyses showed significant within-person influences of mood dimensions on non-exercise activity, yielding distinct sizes and directions of effects for valence, energetic arousal and calmness. Specifically, valence was positively related to non-exercise activity within persons in the 10 min following a particular mood assessment, thus confirming hypothesis I. However, the effect of valence on non-exercise activity was small and the analyses of the other 10-min intervals of non-exercise activity up to 300 min after the mood assessments revealed non-significant effects close to zero. At first sight, this is surprising because three of six existing studies on within-person effects of mood on physical activity found positive affect to significantly influence subsequent physical activity (Liao et al., 2015). However, a closer look at these studies revealed that their results were limited regarding the influence of valence on non-exercise activity. In particular, Carels et al. (2007) were interested in predicting the initiation of self-reported bouts of exercise in obese adults throughout the day by morning mood, and Dunton et al. (2009) investigated a sample of adults (*n* = 23) aged above 50 years and also used self-reported levels of physical activity. Accordingly, both studies examined particular samples using self-reported physical activity, which is known to be only weakly related with objective measures of physical activity (Prince et al., 2008; Adamo et al., 2009). Moreover, Carels et al.'s (2007) study reported on exercise instead of non-exercise activity. Schwerdtfeger et al. (2010) found positive influences of positive affect on physical activity over 12 h using accelerometry in a large sample (*n* = 126). However, they assessed mood using the construct positive activated affect and thus did not differentiate between the mood dimensions valence and energetic arousal. Interestingly, Wichers et al. (2012), who investigated the effects of positive affect on physical activity in a large twin sample (*n* = 504), found weak evidence of positive affect decreasing physical activity. Moreover, only one of the five existing within-subject studies interested in the associations between negative affect and physical activity found significant influences of negative affect on subsequent physical activity, whereas the other studies were not able to show any association (Liao et al., 2015). Therefore, combining our findings with those of existing studies does suggest that valence is not necessarily a strong within-person predictor of non-exercise activity in adults.

As hypothesized (hypothesis II), energetic arousal significantly influenced non-exercise activity within persons. The beta coefficients indicated a strong effect of feeling energetic on non-exercise activity within the 10 min after e-diary assessments. Moreover, energetic arousal significantly influenced all 10-min intervals of non-exercise activity up to 300 min after the e-diary assessment, confirming a robust effect of energetic arousal on non-exercise activity. Our finding is consistent with Dunton et al. (2014), who found feeling less tired and more energetic to be related to subsequent physical activity in 119 children aged eleven to thirteen years. Moreover, Dunton et al. (2009) results indicated a non-significant trend of the effects of feelings of energy and fatigue on non-exercise activity. Schwerdtfeger et al. (2010) found mood, defined as positive activated affect, to positively influence physical activity within their sample. Accordingly, in their study, it remained unclear to which degree positive affect on the one hand and activation on the other hand accounted for this finding. Our results suggest that energetic arousal strongly increases non-exercise activity, whereas the effects of valence are small.

Because existing studies have treated mood as a two-dimensional construct, i.e., mostly investigating how positive and negative affect influence physical activity (Liao et al., 2015), the influence of calmness on physical activity has remained unclear to date. Our results showed significant negative within-person influences of calmness on the 10-min interval of non-exercise activity after mood assessment, thus confirming hypothesis III. In other words, when participants felt calmer, they were less physically active in everyday life. The time course analyses confirmed this finding, showing significant influences of calmness on non-exercise activity noticeably larger than zero for up to 130 min following the e-diary assessment.

## Limitations

Several limitations of our study deserve mentioning. First, due to the accelerated longitudinal design of the URGENCY study with annual follow-ups, our sample consisted of adult participants aged between 18 and 28 years. Accordingly, our results are limited to this restricted age range. Second, as our sample comprised 62% female participants, the gender was unevenly distributed. However, we found no systematic differences in the results when checking for gender effects. Third, we focused our analyses on non-exercise activities (such as daily routines of walking to the office, climbing stairs) thus excluding exercise activities (such as swimming, playing football). Non-exercise activities are most often characterized by short activity duration and low activity intensity (Kanning et al., 2013). Since associations between mood states and physical activity may differ for physical activities with distinct duration and intensity, our results do not reveal, e.g., how mood states are associated with structured exercise activities characterized by high demands of energy consumption. However, as non-exercise activity has been shown to be an important determinant for human health (Healy et al., 2008; Wen et al., 2011), we made the conscious decisions to focus our analyses on non-exercise activity. Further research is needed to investigate within-subject associations between mood states and distinct subcomponents of physical activity. Fourth, as we found significant relationships between mood-dimensions and subsequent non-exercise activity, we interpreted that mood causes changes in non-exercise activity. This assumption of causality is based on the temporal order of mood assessments and examined time frames of non-exercise activity. However, the temporal offset of non-exercise activity relative to the mood ratings constitutes a causal criterion (Susser, 1991) but it does not evidence causality since other existing but hidden causal variables (or mechanisms) that influence both mood and NEA (e.g., circadian rhythms) may have been missed. Reininghaus et al. (2016) propose to conduct ecological momentary intervention studies to verify putative psychological mechanisms. They argue that this kind of approach is suitable to overcome uncertainties of solely investigating several causal criteria therewith reducing the likelihood to miss hidden causal variables. Thus, further studies are needed to evidence causality of mechanisms.

## Conclusion

To the best of our knowledge, this is the first ambulatory assessment study on the topic of within-subject mood effects on non-exercise activity in a large community-based sample of adults over the course of 1 week using repeated real-time and real life assessments of mood, conceptualized as a three-dimensional construct, as well as objectively assessed non-exercise activity. In conclusion, we found differential influences of mood on non-exercise activity. Within participants, feelings of energy and tension strongly increased non-exercise activity in the time following the mood assessment. However, valence showed only small effects on subsequent non-exercise activity. Thus, ambulatory assessment approaches appear suitable to investigate the effects of mood on non-exercise activity. Our results increase the evidence for circular effects of mood and physical activity, thus yielding important insight into the mechanisms maintaining physical activity. These findings may contribute to fostering physically active lifestyles, which are known to be related to high health expectancy.

## Author contributions

MR, HT, IR, HS, AZ, AM, and UE made substantial contributions to the conception and the design of the study. MR and HS acquired data. IR, MR, UE, HT, and AM analyzed and interpreted the data. MR, HT, IR, HS, AZ, AM, and UE were involved in drafting the manuscript and revising it critically for important intellectual content. MR, HT, IR, HS, AZ, AM, and UE have given final approval of this version of the manuscript to be published. MR, HT, IR, HS, AZ, AM, and UE agree to be accountable for all aspects of the work in ensuring that questions related to the accuracy or integrity of any part of this work are appropriately investigated and resolved.

## Funding

The study was supported by the Ministry of Science, Research and the Arts of the State of Baden-Württemberg.

## Disclosures

AL received consultancy fees from: Astra Zeneca, Elsevier, F. Hoffmann-La Roche, Gerson Lehrman Group, Lund-beck foundation, Outcome Europe, Outcome Sciences, Roche Pharma, Servier International, and Thieme Verlag, and lecture fees-including the travel fees-from: Abbott, Astra Zeneca, Aula Congresos, BASF, Groupo Ferrer International, Janssen-Cilag, Lilly Deutschland, LVR Klinikum, Servier Deutschland, Otsuka Pharmaceuticals. The content is solely the responsibility of the authors and was not influenced by any financial relationship with the named organizations.

### Conflict of interest statement

The authors declare that the research was conducted in the absence of any commercial or financial relationships that could be construed as a potential conflict of interest.

## Footnotes

^1^Motorola moto g. http://www.motorola.com/us/products/moto-g. Accessed 07 Feb 2016.

^2^Movisens GmbH. http://www.movisens.com/. Accessed 07 Feb 2016.
